# Superionic lithium mobility in low symmetry Li_7_Si_2_S_7_I polymorph accessed *via* Si_2_S_7_ dimer reorientation

**DOI:** 10.1039/d5sc09834c

**Published:** 2026-06-26

**Authors:** Guopeng Han, Chris M. Collins, Manel Sonni, Luke M. Daniels, Andrij Vasylenko, Ruiyong Chen, Craig M. Robertson, Matthew S. Dyer, John B. Claridge, Matthew J. Rosseinsky

**Affiliations:** a Department of Chemistry, University of Liverpool Crown Street Liverpool L69 7ZD UK; b Leverhulme Research Centre for Functional Materials Design, Materials Innovation Factory, University of Liverpool 51 Oxford Street Liverpool L7 3NY UK m.j.rosseinsky@liverpool.ac.uk

## Abstract

We report the experimental discovery of Li_7_Si_2_S_7_I_0.89_Cl_0.11,_ a triclinic (*P*1̄) analogue of the recently discovered monoclinic (*P*2_1_/*n*) Li_7_Si_2_S_7_I (LSSI), which retains the high computationally predicted Li^+^ ion conductivity of LSSI. Li_7_Si_2_S_7_I_0.89_Cl_0.11_, which is effectively a polymorph of LSSI, maintains the same ordered anion packing based on the packing of spheres in the NiZr intermetallic, however demonstrates a distinct ordering of the Si^4+^ framework-forming cations. While both structures feature Si_2_S_7_ dimers within hexagonal close-packed (hcp) anion motifs, their different arrangement in the triclinic material results in the alternating stacking of silicon-free and silicon-rich layers. Li_7_Si_2_S_7_I_0.89_Cl_0.11_ has an additional Li^+^ position compared to LSSI, sixteen in total, which maintains the large number of redundant low energy pathways favourable for superionic conduction. Thus, Li_7_Si_2_S_7_I_0.89_Cl_0.11_ has a predicted ionic conductivity of 0.019(7) S cm^−1^ derived from molecular dynamics simulation of the experimentally measured structure and a theoretical activation energy for bulk Li^+^ ion transport of 0.16(4) eV, within error of those of monoclinic LSSI. These results demonstrate the structural resilience of the ordered S^2−^/I^−^ anion net to changes in cation positions and crystal system, further exemplifying the ability of the net to afford diverse low barrier Li^+^ ion transport pathways and thus generate a predicted superionic conductivity. Polymorphism is generally thought to have a profound impact on ion transport, however the computational results here suggest that there are privileged anion frameworks with an intrinsic robustness to changes in cation distribution where superionic transport can persist.

## Abstract 2

The experimental structure of Li_7_Si_2_S_7_I_0.89_Cl_0.11_, a triclinic (*P*1̄) analogue of superionic Li_7_Si_2_S_7_I with distinct Si^4+^ ordering, is reported. Li_7_Si_2_S_7_I_0.89_Cl_0.11_ has a predicted conductivity of 0.019(7) S cm^−1^ and activation energy of 0.16(4) eV.

## Graphical abstract

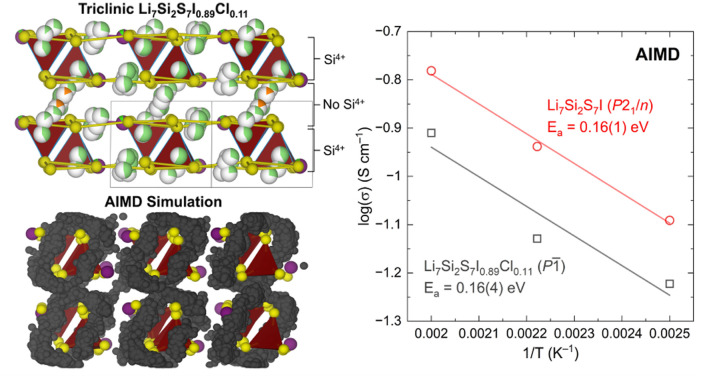

## Introduction

Solid-state electrolytes have gained significant attention as a safer and potentially higher-performance alternative to liquid electrolytes in next-generation battery technologies.^1–5^ The minimisation of Li^+^ coordination changes along the ion conduction pathway within the structure, particularly site energy equivalency provided by similar tetrahedral environments to reduce barriers for transport, is proposed as critical to achieving high Li^+^ ion conductivity in the solid state.^6–8^ This understanding is applied to the few established state-of-the-art solid electrolytes that exhibit superionic (>10^−2^ S cm^−1^) Li^+^ conductivities comparable with those of liquid electrolytes, such as Li_10_GeP_2_S_12_ (LGPS)^9^ type materials,^10,11^ Li_7_P_3_S_11_,^12,13^ and argyrodites.^14,15^ Recently, the superionic conductor Li_7_Si_2_S_7_I (LSSI) which represents a new structural family was discovered using a workflow integrating machine learning and structure prediction.^16^ In contrast to the well-established LGPS and argyrodite structures, LSSI possesses a unique anion packing based on that of the intermetallic NiZr but with a distinct decoration pattern. This ordered S/I array creates a diverse range of interstitial sites available for cation occupation, and offers a wide range of lithium-ion pathways with low energy barriers that enable rapid lithium-ion conduction resulting in a high room temperature ionic conductivity of 1.01(4) × 10^−2^ S cm^−1^. Subsequent substitutional optimisation of LSSI (Li_7_Si_2−*x*_Ge_*x*_S_7_I where *x* ≤ 1.2) further enhanced the Li^+^ site disorder.^17^ This isovalent substitution offers control over the Li^+^ environment size and stabilizes the property-determining disorder to low temperature, yielding enhanced Li^+^ transport in highly substituted (*x* = 1.0–1.2) materials that is comparable to the best solid-state electrolytes. The combination of multiple anions in the structure of LSSI provides additional scope for substitutional optimisation that extends beyond the cations, a well-established method for property control in both argyrodite and LGPS-type materials.^10,11,15,18–20^ Thus, LSSI presents a platform for exploratory substitutional chemistry directed towards further performance enhancement similar to the initial discoveries of argyrodite and LGPS, and their subsequent compositional optimisation to yield the current state-of-the-art materials such as Li_6.6_Si_0.6_Sb_0.4_S_5_I and Li_9.54_[Si_0.6_Ge_0.4_]_1.74_P_1.44_S_11.1_Br_0.3_O_0.6_.^10,21^

In this study, we explore the substitution of I^−^ for Cl^−^ and discover triclinic (*P*1̄) Li_7_Si_2_S_7_I_0.89_Cl_0.11_, a polymorph of monoclinic (*P*2_1_/*n*) Li_7_Si_2_S_7_I, under specific crystal growth conditions. Sub-solidus investigation into the solid solution of Li_7_Si_2_S_7_I_1−*x*_Cl_*x*_ revealed that only slight substitution of I^−^ for Cl^−^ is possible in the monoclinic *P*2_1_/*n* form of LSSI. The structure of triclinic Li_7_Si_2_S_7_I_0.89_Cl_0.11_ is solved by single crystal X-ray diffraction, revealing an ordering of the framework-forming Si_2_S_7_ dimers that is distinct from LSSI. Theoretical assessment of ionic conductivity *via ab initio* molecular dynamics (AIMD) simulations demonstrates that Li_7_Si_2_S_7_I_0.89_Cl_0.11_ retains superionic mobility despite the reduction in symmetry and changes to framework-forming cation positions.

## Results and discussion

The direct substitution of I^−^ for Cl^−^ in Li_7_Si_2_S_7_I_1−*x*_Cl_*x*_ is explored *via* single crystal growth procedures which utilised a range of LiCl–LiI eutectic fluxes (Supporting Information). Crystal growth with a 1 : 1 molar ratio of Li_2_S and SiS_2_ at 773 K in a 35%LiCl–65%LiI flux within a vacuum-sealed silica ampoule afforded a mixture of LiCl, LiI, and Li_2_SiS_3_, including single crystals shown by SEM-EDX to contain both iodine and chlorine, with compositional analysis of multiple crystals affording the composition Si_2.16(13)_S_6.97(11)_I_0.83(16)_Cl_0.2(1)_ (Fig. S1). Single crystal X-ray diffraction demonstrates that these chloride-containing crystals adopt a different structure from LSSI in the triclinic *P*1̄ space group. The *P*1̄ symmetry is retained over the temperature range 100–300 K (SI, Tables S2–S5). The triclinic structure shares the same anion packing as monoclinic LSSI. Refinement reveals reduced electron density on the iodide site, in contrast to the full occupancy found in LSSI itself, consistent with substitution of the chloride indicated by SEM-EDX at that site, leading to a refined composition of Li_7_Si_2_S_7_I_0.8873(17)_Cl_0.1127(17)_.

**Fig. 1:**
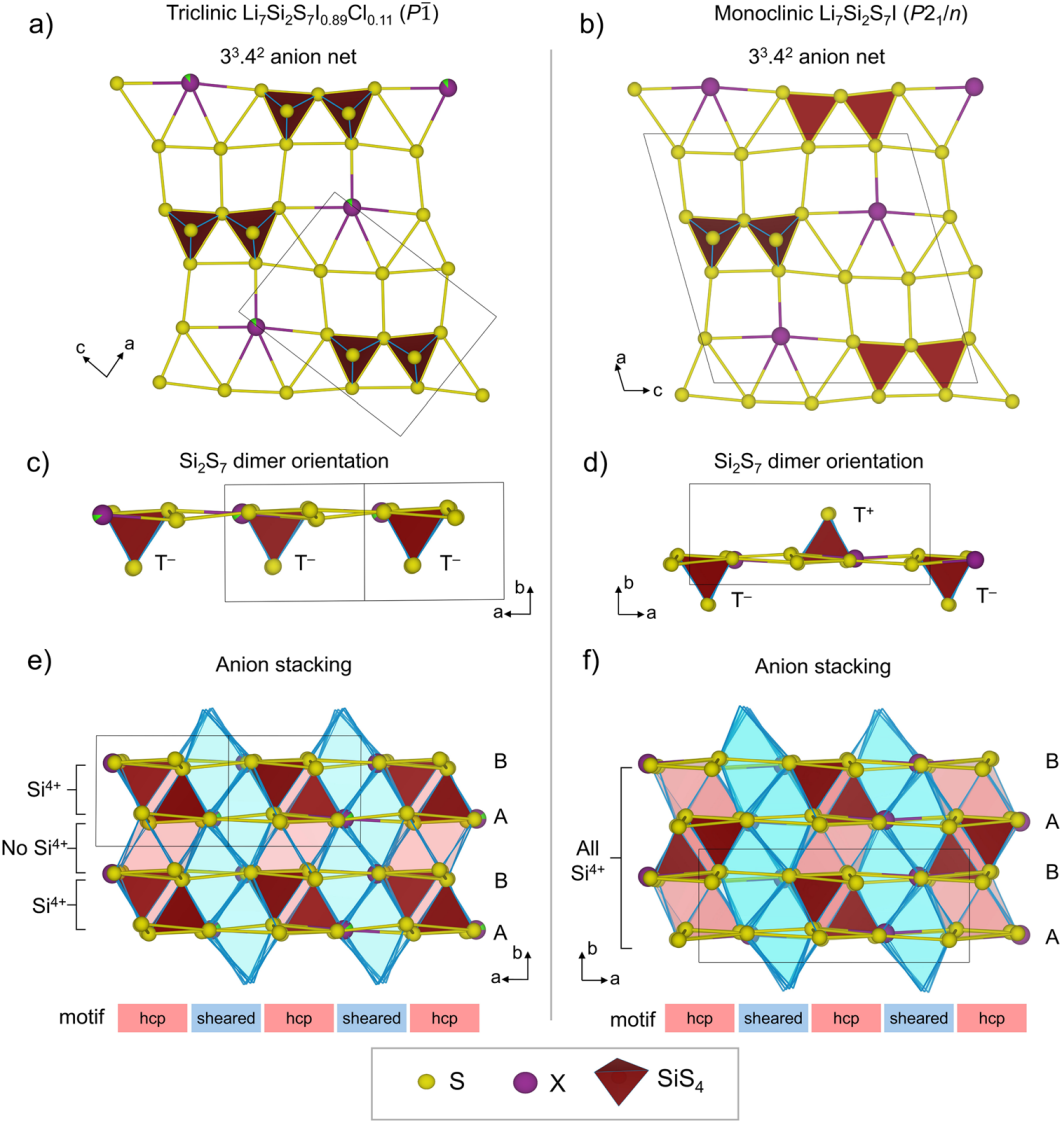
Comparison of anion packing, Si_2_S_7_ dimer arrangement and orientation in triclinic Li_7_Si_2_S_7_I_0.89_Cl_0.11_ and monoclinic Li_7_Si_2_S_7_I. In both (a) Li_7_Si_2_S_7_I_0.89_Cl_0.11_ and (b) Li_7_Si_2_S_7_I, the S^2−^ (yellow) and X^−^ (purple) anions are ordered in 3^3^.4^2^ nets, which stack in an ABAB sequence along *b* to generate hcp (red shading) and sheared fcc-like (blue shading) motifs along the *a* direction shown in (e) and (f). The hcp motifs in both materials accommodate the Si_2_S_7_ tetrahedral dimers (brown) which are arranged on the net in the same way but are oriented differently. In Li_7_Si_2_S_7_I_0.89_Cl_0.11_ the dimers orient (c) below the plane of the anion net (T^−^ tetrahedral environments), compared to the (d) occupancy of both T^+^ and T^−^ tetrahedral environments which are above and below the plane of the anion net in monoclinic LSSI. This distinct Si_2_S_7_ dimer arrangement in triclinic Li_7_Si_2_S_7_I_0.89_Cl_0.11_ generates alternating Si^4+^-free and Si^4+^-rich layers stacked along the *b* axis shown in (e), compared to Si^4+^ environments that are (f) evenly distributed within all layers along *b* in LSSI.

The packing of the S^2−^ and I^−^/Cl^−^ in triclinic Li_7_Si_2_S_7_I_0.89_Cl_0.11_ is based on the 3^3^.4^2^ semiregular net observed in the binary intermetallic NiZr (Fig. 1a). These anion layers stack in an ABAB sequence, generating the same alternating combination of hexagonal close-packed (hcp) and sheared face-centered cubic (fcc)-like anion motifs as observed in monoclinic LSSI (Fig. 1), which define a diverse array of interstitial sites available for cation occupancy in triclinic Li_7_Si_2_S_7_I_0.89_Cl_0.11_. The two Si^4+^ cations do not coordinate to the halide site and occupy adjacent tetrahedral 2*i* sites within the hcp motifs that are solely coordinated by the higher charged S^2−^ to form Si_2_S_7_ dimers. This motif is maintained from the structure of monoclinic LSSI, however, the locations of the framework forming Si^4+^ cations within the hcp fragment of the structures are different in the two symmetries. In triclinic Li_7_Si_2_S_7_I_0.89_Cl_0.11_, the neighbouring Si_2_S_7_ dimers in each anion layer are uniformly oriented occupying the T^−^ sites (Fig. 1c). This is in contrast to the two opposite orientations of Si_2_S_7_ dimers in the monoclinic structure (Fig. 1d) which alternate along the *a* direction between T^+^ and T^−^ tetrahedral environments *i.e.,* with the Si–S vector to the apex of the tetrahedron alternating in orientation. This distinct Si_2_S_7_ dimer arrangement in the structure of Li_7_Si_2_S_7_I_0.89_Cl_0.11_ results in alternating Si^4+^-free and Si^4+^-rich layers stacked along *b* (Fig. 1e), whereas the Si_2_S_7_ dimers are distributed evenly in the layers along *b* in monoclinic LSSI (Fig. 1f).

**Fig. 2:**
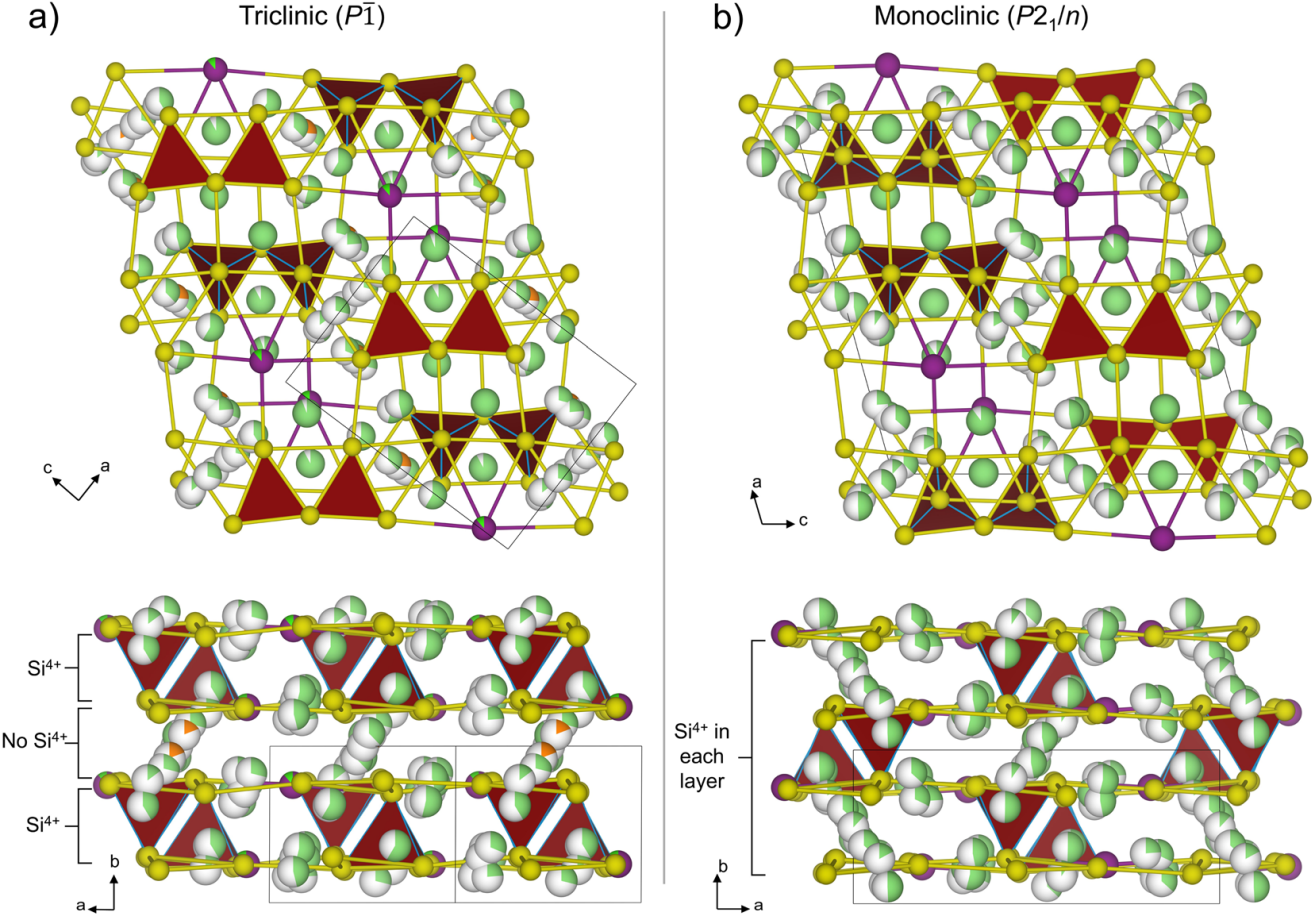
Crystal structures of (a) triclinic Li_7_Si_2_S_7_I_0.89_Cl_0.11_ and (b) monoclinic LSSI at 300 K with lithium sites shown as partially occupied green spheres viewed along the *b* and *c* directions. The partially occupied orange spheres visible in (a) represent the new Li^+^ positions of triclinic Li_7_Si_2_S_7_I_0.89_Cl_0.11_ distinct to those in monoclinic LSSI.

The distinct arrangements of Si_2_S_7_ dimers and the substitutionally disordered I^−^/Cl^−^ site directs the Li^+^ distribution in triclinic Li_7_Si_2_S_7_I_0.89_Cl_0.11_ (Fig. 2). Notably, triclinic Li_7_Si_2_S_7_I_0.89_Cl_0.11_ has sixteen distinct Li^+^ sites within the structure at 300 K, which is the same number as Ge^4+^ substituted Li_7_Si_0.88_Ge_1.12_S_7_I and one more than monoclinic LSSI at the same temperature. The site types occupied by Li^+^ in all three materials are very similar (Fig. 2), consisting of tetrahedral S_3_X and octahedral S_3_X_3_, S_5_X, and S_6_ environments. There are, however, subtle differences between the three materials in the number of each occupied site type (Table S6). Unlike Li_7_Si_0.88_Ge_1.12_S_7_I in which the additional (sixteenth) Li^+^ site occupies an S_6_ octahedral environment within the hcp motif, the additional (sixteenth) site in Li_7_Si_2_S_7_I_0.89_Cl_0.11_ resides within an S_5_X environment in the sheared fcc-like motif. Thus, Li_7_Si_2_S_7_I_0.89_Cl_0.11_ has the same number of occupied Li^+^ sites in the hcp motif as monoclinic LSSI (eight) but one fewer than Li_7_Si_0.88_Ge_1.12_S_7_I (nine), and one more Li^+^ site (eight) occupied in the sheared fcc-like motif compared to both LSSI and Li_7_Si_0.88_Ge_1.12_S_7_I (seven).

**Fig. 3:**
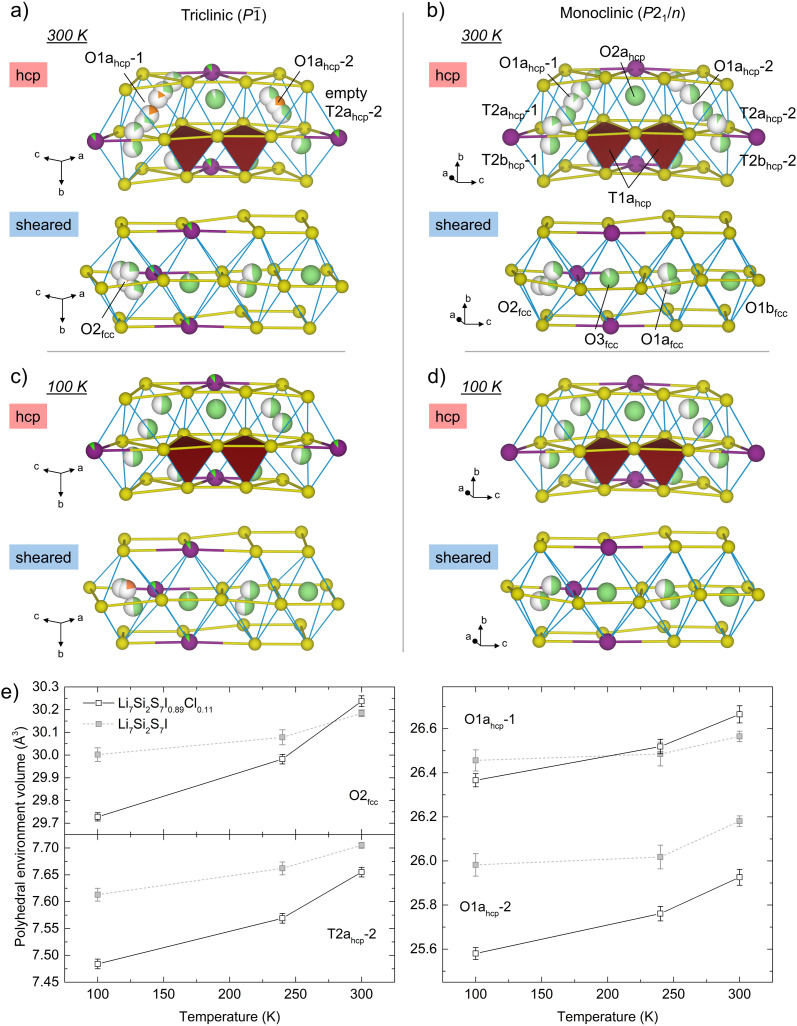
The Li^+^ distribution in hcp and sheared fcc motifs of (a and c) triclinic Li_7_Si_2_S_7_I_0.89_Cl_0.11_ and (b and d) monoclinic LSSI at 300 K and 100 K with lithium sites shown as partially occupied green spheres. The partially occupied orange spheres visible in (a) and (c) represent the new Li^+^ positions in triclinic Li_7_Si_2_S_7_I_0.89_Cl_0.11_ distinct from those observed in monoclinic LSSI. The tetrahedral void T2a_hcp_-2, partially occupied by Li^+^ in monoclinic LSSI, is unoccupied in triclinic Li_7_Si_2_S_7_I_0.89_Cl_0.11_. Similar to monoclinic LSSI, triclinic Li_7_Si_2_S_7_I_0.89_Cl_0.11_ undergoes an isostructural phase transition in which four of the sixteen Li^+^ sites observed at 300 K become unoccupied at 100 K. The volumes of particular environments within monoclinic LSSI and triclinic Li_7_Si_2_S_7_I_0.89_Cl_0.11_ as a function of temperature are compared in (e).

The exact Li^+^ site distributions within the hcp and sheared fcc-like motifs of triclinic Li_7_Si_2_S_7_I_0.89_Cl_0.11_ have slight differences from those of monoclinic LSSI, which are associated with changes in the volumes of certain cation coordination environments following the partial substitution of I^−^ for Cl^−^ (Tables S7 and S8).^22^ Firstly, one of the partially occupied Li^+^ sites observed in the hcp motif of monoclinic LSSI is unoccupied in Li_7_Si_2_S_7_I_0.89_Cl_0.11_. The S_3_I-defined tetrahedra is partially occupied by Li^+^ in monoclinic LSSI, however it is unoccupied in triclinic Li_7_Si_2_S_7_I_0.89_Cl_0.11_ (labelled as T2a_hcp_-2 in Fig. 3a and b) as a result of the tetrahedral volume decreasing from 7.7049(18) Å^3^ to 7.655(3) Å^3^, respectively. Secondly, a new distinct Li^+^ position is observed in the hcp motif of Li_7_Si_2_S_7_I_0.89_Cl_0.11_ which is unoccupied in monoclinic LSSI. This site is located on a 1*b* Wyckoff position at the centre of an S_6_ octahedron in the hcp motif (site shown in orange and labelled as O1a_hcp_-2 in Fig. 3a). Despite the volume of this octahedron decreasing slightly from 26.180(8) Å^3^ in monoclinic LSSI to 25.926(12) Å^3^ in triclinic Li_7_Si_2_S_7_I_0.89_Cl_0.11_, the occupation of this new site in Li_7_Si_2_S_7_I_0.89_Cl_0.11_ results from the redistribution of Li^+^ from the now unoccupied neighbouring T2a_hcp_-2 environment described above. Thirdly, the Li^+^ site that occupies a 2*c* Wyckoff position in the other S_6_ octahedron (labelled as O1a_hcp_-1 in Fig. 3a and b) of the hcp motif in monoclinic LSSI splits onto a 2*i* Wyckoff position in triclinic Li_7_Si_2_S_7_I_0.89_Cl_0.11_ such that it coordinates to four S^2−^ anions (site shown in orange in Fig. 3a). This is a result of the O1a_hcp_-1 S_6_ octahedron volume increasing from 26.565(8) Å^3^ to 26.665(13) Å^3^, respectively. Thus, aside from the unoccupied T2a_hcp_-2 site, the Li^+^ site distribution within the hcp motif of Li_7_Si_2_S_7_I_0.89_Cl_0.11_ is more closely comparable to that seen in Li_7_Si_0.88_Ge_1.12_S_7_I rather than LSSI. Finally, the second additional Li^+^ site occupied in Li_7_Si_2_S_7_I_0.89_Cl_0.11_ that is not occupied in LSSI or Li_7_Si_0.88_Ge_1.12_S_7_I is located within the sheared fcc-like motif on a 2*i* Wyckoff position in an S_5_X octahedron (labelled as O2_fcc_ in Fig. 3a). This is enabled by a volume expansion of the O2_fcc_ octahedron from 30.184(5) Å^3^ in monoclinic LSSI to 30.237(8) Å^3^ in triclinic Li_7_Si_2_S_7_I_0.89_Cl_0.11_. The volumes of the environments discussed above in monoclinic LSSI and triclinic Li_7_Si_2_S_7_I_0.89_Cl_0.11_ are compared in Fig. 3e. The majority of the Li^+^ environments that incorporate the mixed I^−^/Cl^−^ position in Li_7_Si_2_S_7_I_0.89_Cl_0.11_ decrease in volume (Tables S7 and S8) relative to monoclinic LSSI as a result of the reduced average ionic radius of that I^−^/Cl^−^ position (Table S9) . The occupancies of all Li^+^ sites observed in triclinic Li_7_Si_2_S_7_I_0.89_Cl_0.11_ at 300 K are summarized in Table S3.

Triclinic Li_7_Si_2_S_7_I_0.89_Cl_0.11_ undergoes a similar isostructural phase transition to that observed in monoclinic LSSI with the depopulation of four Li^+^ sites at low temperatures (Fig. 3c and d). Two of the sixteen Li^+^ sites occupied at 300 K in Li_7_Si_2_S_7_I_0.89_Cl_0.11_ become unoccupied at 240 K (S_6_-defined O1a_hcp_-1 and S_3_X-defined T2a_hcp_-1), and a further two sites are unoccupied at 100 K (S_6_-defined O1a_hcp_-2 and S_5_X-defined O2_fcc_). Triclinic Li_7_Si_2_S_7_I_0.89_Cl_0.11_ retains three Li^+^ positions in the S_5_X-defined O2_fcc_ octahedron at 100 K (Fig. 3c), which is one more than monoclinic LSSI at the same temperature (Fig. 3d). Precise tuning of composition *via* volume-controlling substitution, such as the Ge^4+^ substitution for Si^4+^ demonstrated for monoclinic LSSI, may present a promising strategy for optimisation of the property-determining Li^+^ distribution within the triclinic structure.

Though crystal growth of triclinic Li_7_Si_2_S_7_I_0.89_Cl_0.11_ was successful, despite extensive efforts it was not possible to isolate a high purity powder sample preventing the experimental measurement of ionic conductivity, even with the compositional guidance provided by SEM-EDX. Triclinic Li_7_Si_2_S_7_I_0.89_Cl_0.11_ was not observed in any bulk powder reactions that were performed at a range of different compositions and synthesis conditions (Table S1). In all cases, the monoclinic phase of LSSI was observed, and the phase purity decreased in both compositions with increased Cl content, or reactions run at higher synthesis temperatures (Fig. S3). Thus, the synthesis of triclinic Li_7_Si_2_S_7_I_0.89_Cl_0.11_ is more complicated than targeting a specific composition alone. We note that the exact crystal growth procedure in which triclinic Li_7_Si_2_S_7_I_0.89_Cl_0.11_ is observed differs from the procedure used for monoclinic LSSI, which utilised elemental S as the flux, as well as different growth temperatures and durations. Indeed, crystals of triclinic Li_7_Si_2_S_7_I_0.89_Cl_0.11_ were obtained only from the syntheses which utilised the 35%LiCl–65%LiI eutectic flux (SI); triclinic Li_7_Si_2_S_7_I_0.89_Cl_0.11_ was not observed in any elemental S flux growths that yield monoclinic LSSI, or even Li_7_Si_2−*x*_Ge_*x*_S_7_I. This indicates that triclinic Li_7_Si_2_S_7_I_0.89_Cl_0.11_ may be accessible only under a narrow range of synthesis conditions and that competition with monoclinic LSSI makes experimental isolation as a bulk powder challenging.

**Fig. 4:**
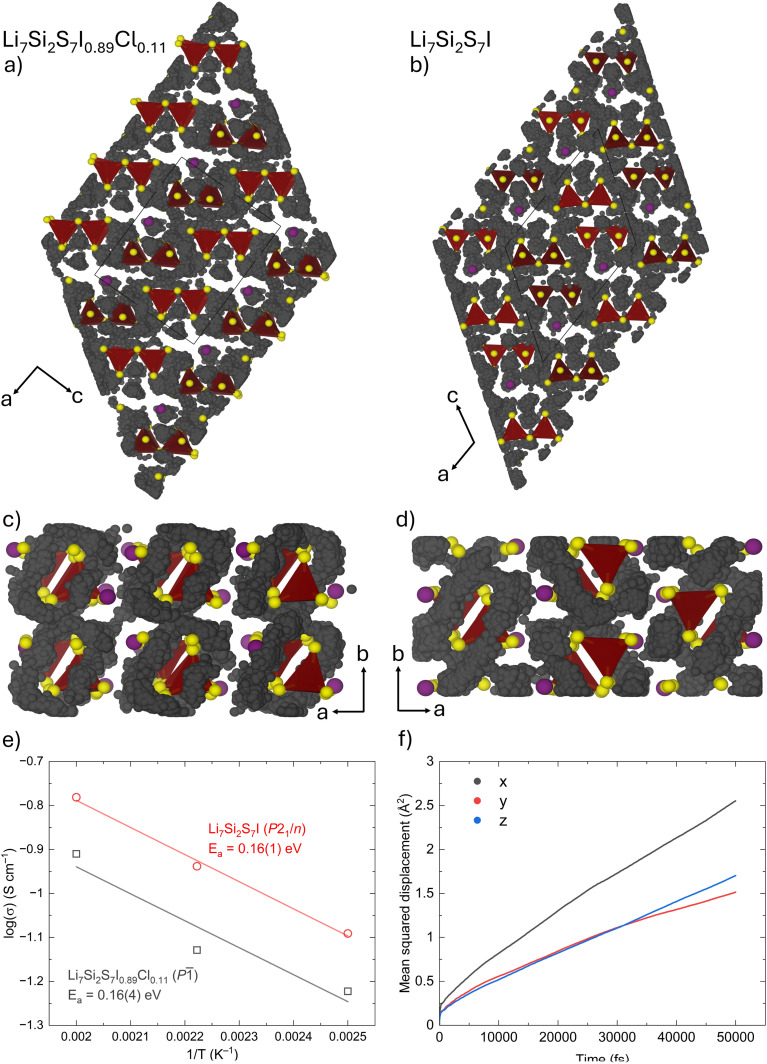
AIMD simulation of (a) triclinic Li_7_Si_2_S_7_I_0.89_Cl_0.11_ (model composition Li_7_Si_2_S_7_I) and (b) monoclinic LSSI. Black points indicate the calculated Li^+^ positions taken at time intervals of 0.5 ps from the 250 ps AIMD simulation at 500 K for Li_7_Si_2_S_7_I_0.89_Cl_0.11_, and 0.5 ps intervals from a 300 ps AIMD simulation at 500 K for LSSI.^16^ Projections along *c* for (c) Li_7_Si_2_S_7_I_0.89_Cl_0.11_ and (d) LSSI. (e) Comparison of computed ionic conductivities (log(*σ*)) for Li_7_Si_2_S_7_I_0.89_Cl_0.11_ and LSSI, with extracted activation energies. (f) Mean squared displacements (msd) of the three crystallographic axes in Li_7_Si_2_S_7_I_0.89_Cl_0.11_ at 500 K. Comparable gradients for all three traces indicate that Li_7_Si_2_S_7_I_0.89_Cl_0.11_ is a 3D Li^+^ ion conductor. Panel (b) is reproduced or adapted with permission from ref. 16. Copyright 2025 The American Association for the Advancement of Science.

In the absence of experimental measurements of the conductivity, the Li^+^ ion transport properties of triclinic Li_7_Si_2_S_7_I_0.89_Cl_0.11_ were thus examined by *ab initio* molecular dynamics (AIMD) simulations^23–29^ that used the experimentally determined crystal structure, where the framework-forming Si, S and halide sites that define the pathways within which the heavily disordered Li move are well-defined crystallographically: the MD simulations naturally account for the disorder in the highly mobile Li. These simulations demonstrated that the overall ion transport is highly comparable to that of monoclinic LSSI (Fig. 4a–d). Specifically, the calculated diffusion coefficients along each crystallographic axis are comparable with those of LSSI and agree within a factor of two indicating 3D Li^+^ mobility which results from the vast array of migration pathways provided by the structure (Fig. 4f), as in LSSI. A theoretical activation energy of 0.16(4) eV is extracted for Li_7_Si_2_S_7_I_0.89_Cl_0.11_ from the mean squared displacements across simulations at 400–500 K, and a computed bulk ionic conductivity of 0.019(7) S cm^−1^ extrapolated to 300 K (Fig. 4e). These values are within error of those obtained from equivalent AIMD simulations on monoclinic LSSI with an activation energy of 0.16(1) eV and calculated conductivity of 0.023(9) S cm^−1^. These simulations further support that the intermetallic-derived anion framework of LSSI enables rapid Li^+^ ion transport throughout the structure, and that the Li mobility pathways and associated computed high performance is retained despite changes to both the crystal system and arrangement of the framework-forming Si_2_S_7_ dimers.

## Conclusions

Li_7_Si_2_S_7_I_0.89_Cl_0.11_ is effectively a triclinic polymorph of LSSI. It demonstrates that the anion framework originally observed in Li_7_Si_2_S_7_I, derived from the sphere packings within the NiZr intermetallic, can accommodate changes in both crystal system and the location of framework-forming Si^4+^ cations. The ordered arrangement of S^2−^ and X^−^ anions from LSSI is retained, while the substitution at the halide site generates distinct orientations of Si_2_S_7_ dimers that produce alternating arrangements of silicon-free and silicon-rich layers along the stacking axis. The ability to vary the orientation of Si_2_S_7_ dimers is an additional degree of freedom for the control of structure and properties beyond those of the argyrodite and LGPS families, whose structures are defined by isolated tetrahedral MS_4_ positions. Triclinic Li_7_Si_2_S_7_I_0.89_Cl_0.11_ has sixteen distinct Li^+^ sites in total, one more than LSSI, and retains a superionic computed conductivity and low computed activation energy. Polymorphism is known to significantly impact ion transport in other Li^+^ conductor families, *e.g.,* the Li^+^ site ordering observed in argyrodite and thio-LISICON materials.^30–34^ The LSSI ordered S^2−^ and X^−^ anion framework maintains multiple low energy pathways from AIMD simulations for lithium motion despite significant changes in the framework-forming cation distribution associated with the reduction in symmetry from monoclinic to triclinic. This persistence of predicted high ion mobility from simulation in the presence of polymorphism suggests that there are privileged anion frameworks that can tolerate changes in cation distribution while retaining superionic transport.

## Author contributions

GH developed synthetic methodology for isolation of the new phase. GH solved the crystal structure with support from CMR, LMD and JBC. CMC performed AIMD simulations to predict ion transport behaviour with support of MSD. MS conducted measurements for elemental analysis. MJR directed the research and acquired funding. GH and LMD composed the initial draft of the manuscript. All authors contributed to the review and editing of the manuscript.

## Conflicts of interest

The authors declare no conflicts of interest.

## Supplementary Material

SC-OLF-D5SC09834C-s001

SC-OLF-D5SC09834C-s002

## Data Availability

The data that support the findings of this research will be made openly available *via* the University of Liverpool open research platform Liverpool Elements on acceptance of the publication. CCDC 2429627 (100 K), 2429628 (240 K), and 2429626 (300 K) contain the supplementary crystallographic data for this paper.^35*a*–*c*^ Supplementary information (SI) is available. See DOI: https://doi.org/10.1039/d5sc09834c.
