# Supplementary material for: Superionic lithium mobility in low symmetry Li7Si2S7I polymorph accessed via Si2S7 dimer reorientation
Source: Chem Sci. 2026 Jun 26;17(33):15875–82. doi: 10.1039/d5sc09834c (PMC13345648; doi:10.1039/d5sc09834c)
Supplement: Supplementary file 1 [file SC-017-D5SC09834C-s001.pdf]

Supplementary Information (SI) for Chemical Science.
This journal is © The Royal Society of Chemistry 2026

Supporting Information

for

Superionic Lithium Mobility in Low Symmetry $Li_7Si_2S_7I$ Polymorph Accessed via Si_2S_7 Dimer Reorientation

Guopeng Han,[a] Chris M. Collins,[a,b] Manel Sonni,[a] Luke M. Daniels,[a] Andrij Vasylenko,[a] Ruiyong Chen,[a] Craig M. Roberston,[a] Matthew S. Dyer,[a,b] John B. Claridge,[a,b] and Matthew J. Rosseinsky*[a,b]

[a] Department of Chemistry, University of Liverpool, Crown Street, Liverpool, L69 7ZD, United Kingdom

[b] Leverhulme Research Centre for Functional Materials Design, Materials Innovation Factory, University of Liverpool, 51 Oxford Street, Liverpool, L7 3NY, United Kingdom

* Corresponding Author: M.J.Rosseinsky@liverpool.ac.uk

Computational methods

Obtaining the starting model for ab-initio molecular dynamics

To obtain the starting structure for *ab initio* molecular dynamics simulations, the experimental structure of $Li_7Si_2S_7I_{0.89}Cl_{0.11}$ at 300 K was used. The mixed I/Cl site was set to be fully occupied by I. For the disordered Li sites, where sites overlap, one site was arbitrarily selected for the model, with the remaining sites with greater than 0.9 occupancy, they were set to be fully occupied by one Li. This thus yields an initial model with the composition $Li_7Si_2S_7I$ but based on the experimental structure of $Li_7Si_2S_7I_{0.89}Cl_{0.11}$, containing two formula units.

To further explore the configuration of Li within the structure, the code ChemDASH[1] was used to iteratively test modifications to the Li sub-structure, controlled by a Monte-Carlo search. This was run for 500 steps, only allow Li atoms to be swapped within the structure. After each step was taken the structure was optimised to a local minimum using the density functional theory (DFT) code VASP[2], using PBE[3] pseudo potentials. Structures were relaxed until either forces were below 0.05 eV $Å^{-1}$, or a total of 1,050 ionic steps had been attempted, with each electronic step converged to 1×10^{-4} eV, with automatic k-point sampling controlled by the KSPACING parameter in VASP, with a setting of 0.3 and a plane wave cutoff energy of 650 eV. The lowest energy structure was then carried forwards as the start point for the molecular dynamics simulation detailed in the next section.

Ab-initio molecular dynamics (AIMD)

To adequately gather statistics on Li motion, a $3 \times 2 \times 1$ supercell of the ChemDASH structure was created, containing 204 atoms. The simulation was performed in line with our previous work on LSSI[4] with details restated here for completeness. Prior to the start of the AIMD simulation the structure was re-optimised with Γ-point only k-point sampling, with a plane wave cut-off of 650 eV and until forces were below 0.01 eV $Å^{-1}$.

Fixed cell AIMD simulations were then carried out at three temperatures (400, 450 and 500 K) as follows: the plane wave cutoff energy was reduced to 600 eV, with reciprocal space sampled at the Γ-point only, a timestep of 0.5 fs was used throughout the simulations using PBE pseudo potentials as mentioned above. An initial temperature ramp from 0 K to the target temperature was carried out over 4 ps followed by an equilibration period of 10 ps, with the temperature controlled at each step by velocity scaling. The production runs were then carried out for 250 ps for each temperature using a Nosé thermostat.[5] Diffusion coefficients, Li conductivity and activation energies were then extracted, based on our previous work,[4] outlined in section 1.1.4 of the SI, restated here for completeness:

The mean squared deviation (msd) was computed by splitting each AIMD trajectory (for each temperature, T) into five equal parts (of 50 ps) and then taking the mean value at each time step, t. This is according to the method presented in reference.[6] Calculations for diffusion coefficients, activation barriers to transport and ionic conductivity were performed according to the methods presented in reference.[7] The diffusion coefficient, D_T, for a given temperature, T, is defined by the following equation:

$$D_{\mathrm{T}} = \frac{1}{2dt} < [\Delta r(t)^2] > \qquad \text{Eq. 1}$$

where d is the dimensionality of the ionic conductivity (3D for $Li_7Si_2S_7I$) and $< [\Delta r(t)^2] >$ is the mean squared deviation in Å^2. D_{T} is then determined by plotting the msd vs. t (in fs) and fitting the gradient. In this work, the fit was conducted between 10,000 and 40,000 fs as this removes the initial non-linear part of the plot which arises from atomic motion. Then then yields $6D_{\mathrm{T}}$ in the units Å^2 fs^{-1}, which is then divided by 6 and converted into SI units of m^2 s^{-1}.

Conductivity at temperature, σ_{T}, is then calculated according to the Nernst-Einstein equation:

$$\sigma_{\mathrm{T}} = \frac{\rho z^2 F^2}{RT} D_{\mathrm{T}} \qquad \text{Eq. 2}$$

where ρ is the molar density of Li ions in the DFT model (in mol m^{-3}), which for the unit cell used in AIMD simulation for $Li_7Si_2S_7I_{0.89}Cl_{0.11}$ is 33311.78 mol m^{-3}, z is the Li charge (+1), F is the Faraday constant (96485.33212 C mol^{-1}), R is the molar gas constant (8.314462618 J mol^{-1} K^{-1}), T and D_{T} are the temperature (in K) and the diffusion coefficient (m^2 s^{-1}) as defined above. This equation then yields the conductivity σ_{T} in units of S m^{-1} which is then converted to S cm^{-1}.

The activation energy, E_{A}, is determined by the Arrhenius relationship:

$$D_{\mathrm{T}} = D_0 \exp\left(-\frac{E_{\mathrm{A}}}{k_{\mathrm{B}}T}\right) \qquad \text{Eq. 3}$$

where D_{T} is the diffusion coefficient, D_0 is maximum diffusivity as infinite temperature (in m^2 s^{-1}), k_{B} is the Boltzmann constant (1.38065×10^{-23} J K^{-1}) and T the temperature in K.

This equation is then linearised to:

$$\ln(D_{\mathrm{T}}) = -\frac{E_{\mathrm{A}}}{k_{\mathrm{B}}}\frac{1}{T} + \ln(D_0) \qquad \text{Eq. 4}$$

where E_{A} (in J) is then computed by multiplying the gradient of this plot by $-k_{\mathrm{B}}$, which is then converted into eV. In this work, an E_{A} = 0.16(4) eV is obtained for $Li_7Si_2S_7I_{0.89}Cl_{0.11}$. Using this fitted value for E_{A} and D_0 the 300 K conductivity was extrapolated, and was found to be 0.019(7) S cm^{-1}.

Synthesis and characterization

Materials

Li_2S (99.9%, Thermo Scientific), SiS_2 (99.999%, MSE Supplies), Si (99.998%, SigmaAldrich), S (99.998%, Sigma-Aldrich), LiCl (99.9%, Alfa Aesar) and LiI (99.99%, Sigma-Aldrich) were dried under dynamic vacuum overnight before use. The quartz ampoule containing graphite crucibles were flame-dried under dynamic vacuum (< 10^{-4} mbar) before use. Precursors and resulting powders were handled in an Ar-filled glovebox (O_2 < 0.5 ppm, H_2O < 0.1 ppm).

Crystal growth of triclinic $Li_7Si_2S_7I_{0.89}Cl_{0.11}$

Crystals of triclinic $Li_7Si_2S_7I_{0.89}Cl_{0.11}$ were obtained by the crystallization of Li_2S, SiS_2, and LiI reagents from a eutectic flux of 35%LiCl-65%LiI. The SiS_2 was prepared following the method described previously.[4] The starting materials of Li_2S, SiS_2, LiCl and LiI were weighed with the ratio of 1:1:1:1.86, which were then ground, pelletized, transferred into a graphite crucible and sealed in an evacuated silica ampoule. The ampoule containing the sample was heated to 773 K for 48 hours with a rate of 5 K min^{-1}, followed by a slow cooling to room temperature at a rate of 1 K min^{-1}. The crystals suitable for single-crystal X-ray diffraction (XRD) were visually identified and gently isolated from the resulting crystalline products.

Single crystal X-ray diffraction

A block-like single crystal with dimensions of approximate $50 \times 60 \times 70$ μm^3, identified as a new phase through screening crystals isolated from the sample, was selected for single crystal X-ray diffraction analysis. The crystal was mounted on a Rigaku 007HF Mo rotating anode single crystal diffractometer. To analyse the structure as a function of temperature, data were collected at three different temperatures 100, 240, and 300 K. Data reduction was performed with the CrysAlisPro (Version 171.40_64.53) software.[8] The structures were solved by Intrinsic Phasing method provided by the ShelXT[9] program, and anisotropically refined using least-squares method incorporated in the ShelXL[10] refinement package, interfaced through Olex2 program.[11] The occupancy of all sites was freely and simultaneously anisotropically refined, indicating that all Si^{4+} and S^{2-} sites are all fully occupied and their occupancies were consequently fixed at 1 during the refinements. The I^- and Cl^- share the same crystallographic site with refined occupancy of 0.8873(17)/0.1127(17). The overall Li^+ content was constrained to charge balance against the anion content, yielding the final models with the composition of $Li_7Si_2S_7I_{0.89}Cl_{0.11}$. Trial refinements were used to locate Li^+ positions which were placed on peaks of weak residual electron density observed in Fourier maps followed by occupancy refinement. Final Li^+ positions were assigned only when their refined occupancies were ~10% or higher and their coordination environments were chemically meaningful. Other regions of weak residual electron density, such as peaks located too close to heavier S^{2-} or I^- positions were treated as artefacts possibly introduced by Fourier truncation effects. Tabulated crystallographic data can be found in Table S2. The final refined atomic positions, site occupancy factors, isotropic thermal parameters, anisotropic displacement parameters of each atom, and selected atom distances are summarized in Tables S3−6. Images of structural models were drawn using the program VESTA.[12]

Exploratory synthesis of the triclinic phase in the Li-Si-S-Cl-I phase field

Attempts to synthesize a bulk sample of the triclinic phase were performed with the solid solution compositions of $Li_7Si_2S_7I_{1-x}Cl_x$, where x = 0.05 and 0.1. Following the same synthesis procedure as monoclinic $Li_7Si_2S_7I$ (LSSI), the precursors Li_2S, SiS_2, LiCl and LiI were manually ground with a mortar and pestle, and then pelletized, placed in a graphite crucible and vacuum-sealed in a quartz ampoule. The 6% excess of SiS_2 was used to improve the product purity. The ampoule was heated at 723 K for 4 days with intermediate grinding after two days. The resulting powder was then ground and analysed with X-ray diffraction,

confirming the absence of the triclinic phase (Figure S2). The major phases formed correspond to monoclinic LSSI with a small contribution of the impurity LiI for the composition x = 0.05 (purity: ~98%) and Li_4SiS_4, $LiSiS_3$ and LiI for the composition x = 0.1 (purity: ~88%).

Following the same procedure as above, two other compositions of $Li_{6.9}Si_2S_{6.9}I_{0.9}Cl_{0.2}$ and $Li_{6.72}Si_2S_{6.8}Cl_{0.2}I_{0.92}$ were also explored both at two different temperatures 723 and 773 K. The X-ray diffraction patterns of the resulting powders indicated that the major phase obtained was monoclinic LSSI, with the impurities of LiI, Li_4SiS_4 and $LiSiS_3$. Table S1 and Figure S3 detail the range of conditions explored and the corresponding results.

Crystal growth of $Li_7Si_2S_7I_{0.89}Cl_{0.11}$ was attempted from an elemental S flux with four different molar ratios of LiCl/LiI, namely 10%LiCl-90%LiI, 25%LiCl-75%LiI, 75%LiCl-25%LiI and 100% LiCl. The experiments were carried out at 723 K for the first two compositions and 773 K for the latter two compositions, with the same procedure as used for LSSI and $Li_7Si_{0.88}Ge_{1.12}S_7I$. However, the X-ray diffraction patterns of the resulting samples show that monoclinic LSSI was formed only at the ratio of 10%LiCl-90%LiI, and that all other compositions failed to form LSSI, instead forming Li_4SiS_4 and Li_2SiS_3.

Table S1. Conditions and results of the experimental exploration of Cl substitution in LSSI.

Composition	Condition	Result	wt% of LSSI
$Li_7Si_2S_7I_{0.95}Cl_{0.05}$	723 K for 4 days with an intermediate grinding	monoclinic LSSI and LiI	98%
$Li_7Si_2S_7I_{0.9}Cl_{0.1}$	723 K for 4 days with an intermediate grinding	monoclinic LSSI, LiI, Li_4SiS_4 and $LiSiS_3$	88%
$Li_{6.9}Si_2S_{6.9}I_{0.9}Cl_{0.2}$	723 K for 2 days	monoclinic LSSI, LiI, Li_4SiS_4, $LiSiS_3$ and LiCl	57%
	773 K for 2 days	monoclinic LSSI, LiI, Li_4SiS_4, $LiSiS_3$ and LiCl	47%
$Li_{6.72}Si_2S_{6.8}I_{0.92}Cl_{0.2}$	723 K for 2 days	monoclinic LSSI, LiI, LiCl, Li_4SiS_4 and $LiSiS_3$	54%
	773 K for 2 days	monoclinic LSSI, LiI, LiCl, Li_4SiS_4 and $LiSiS_3$	45%

Powder X-ray diffraction

Routine assessment of sample purity was carried out using Rigaku SmartLab diffractometer with Mo radiation ($K\alpha_1$, λ = 0.7093 Å) in Debye−Scherrer transmission geometry. Synchrotron powder XRD was performed at the I11 beamline at Diamond Light Source (Oxfordshire, U.K.), with an incident wavelength of 0.82473 Å using a wide-angle position sensitive detector. The experiments were performed at room temperature with samples sealed in Ø = 0.5 mm borosilicate glass capillaries.

Elemental analysis

To identify the bulk composition of the triclinic phase, scanning electron microscopy energy-dispersive X-ray (SEM-EDX) elemental analysis was performed using a TESCAN S800 microscope equipped with SDD detector from Oxford Instruments. Powder containing

crystals was dispersed on carbon tape attached to a brass stub. The samples were transported from the glovebox to the SEM microscope using a Quorum transfer holder for air sensitive materials. Data acquisition and analysis were performed using Aztec software. Correction factors were determined by measuring standards for each of the chemical elements. Data were collected from several separate crystals to identify the composition of the sample.

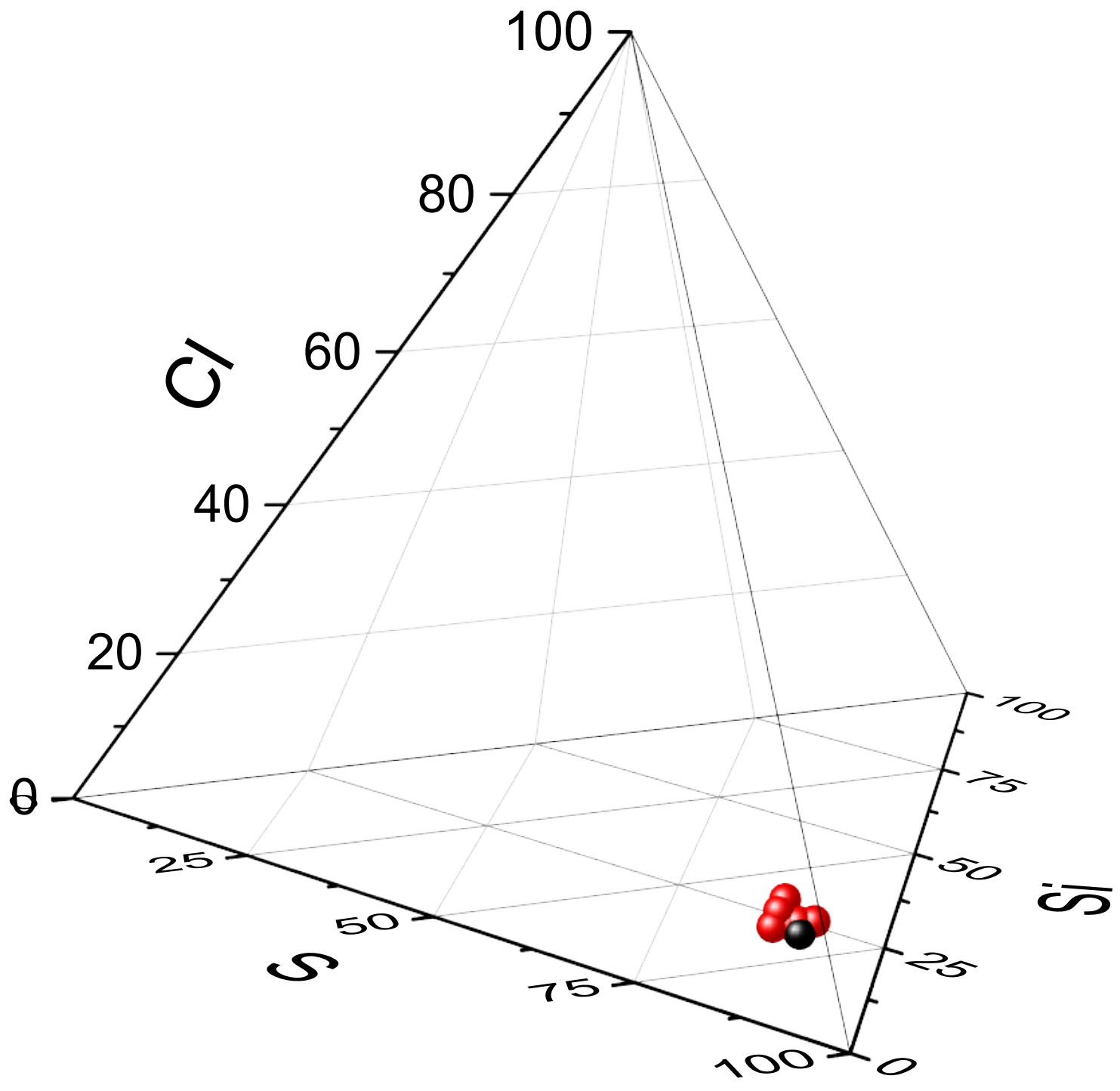

Figure S1. Compositions measured by SEM-EDX (red spheres) obtained from six separate crystal particles of triclinic $Li_7Si_2S_7I_{0.89}Cl_{0.11}$. The average composition is $Si_{2.16(13)}S_{6.97(11)}I_{0.83(16)}Cl_{0.2(1)}$ when normalized to a total anion content of 8, which is consistent with the composition of $Li_7Si_2S_7I_{0.89}Cl_{0.11}$ (black sphere) refined from single crystal X-ray diffraction data.

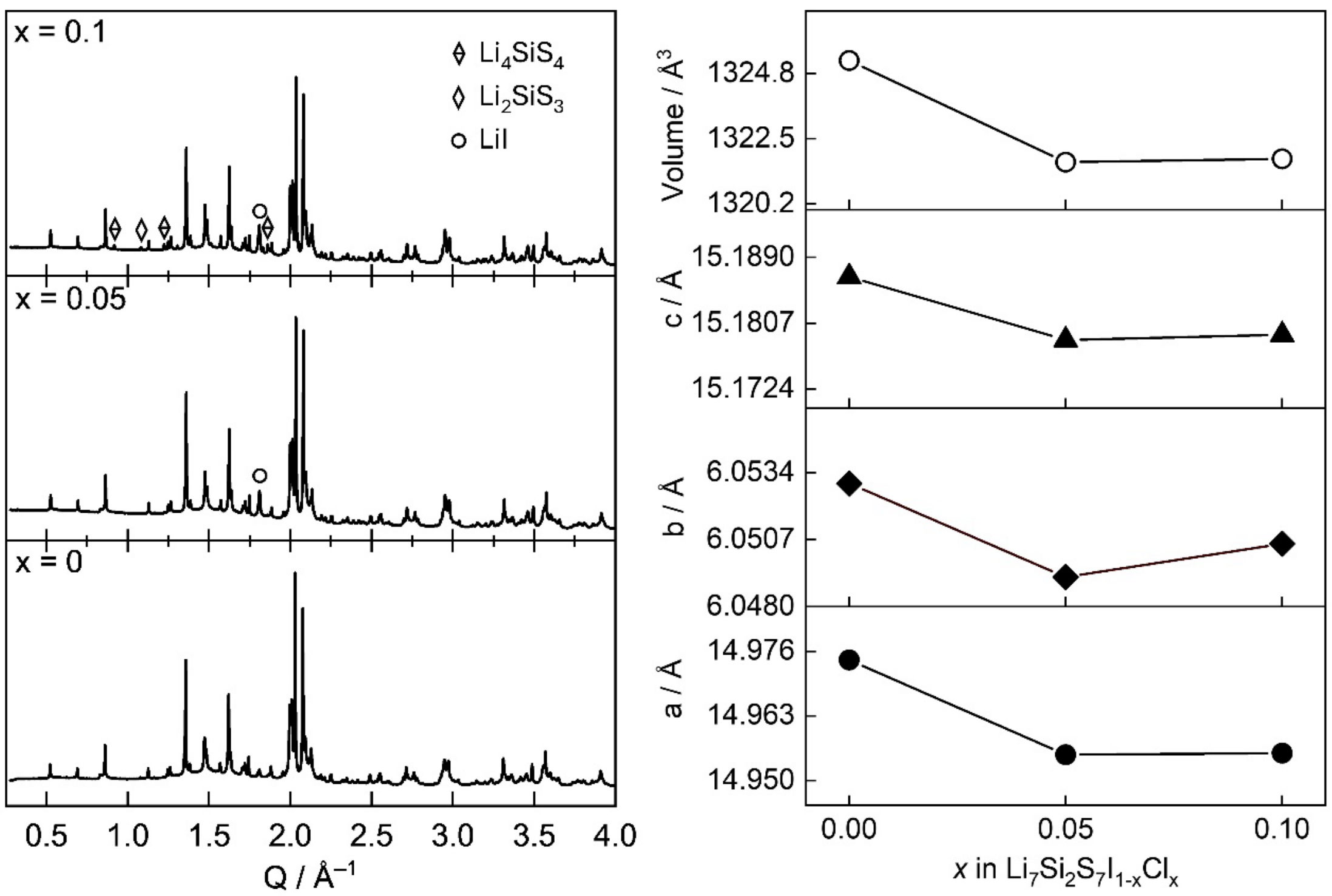

Figure S2. Synchrotron powder X-ray diffraction patterns (beamline I11, Diamond) collected from samples of compositions $Li_7Si_2S_7I_{1-x}Cl_x$, where x = 0.05 and 0.1. b) Variation of refined unit cell parameters and volume as a function of x in $Li_7Si_2S_7I_{1-x}Cl_x$.

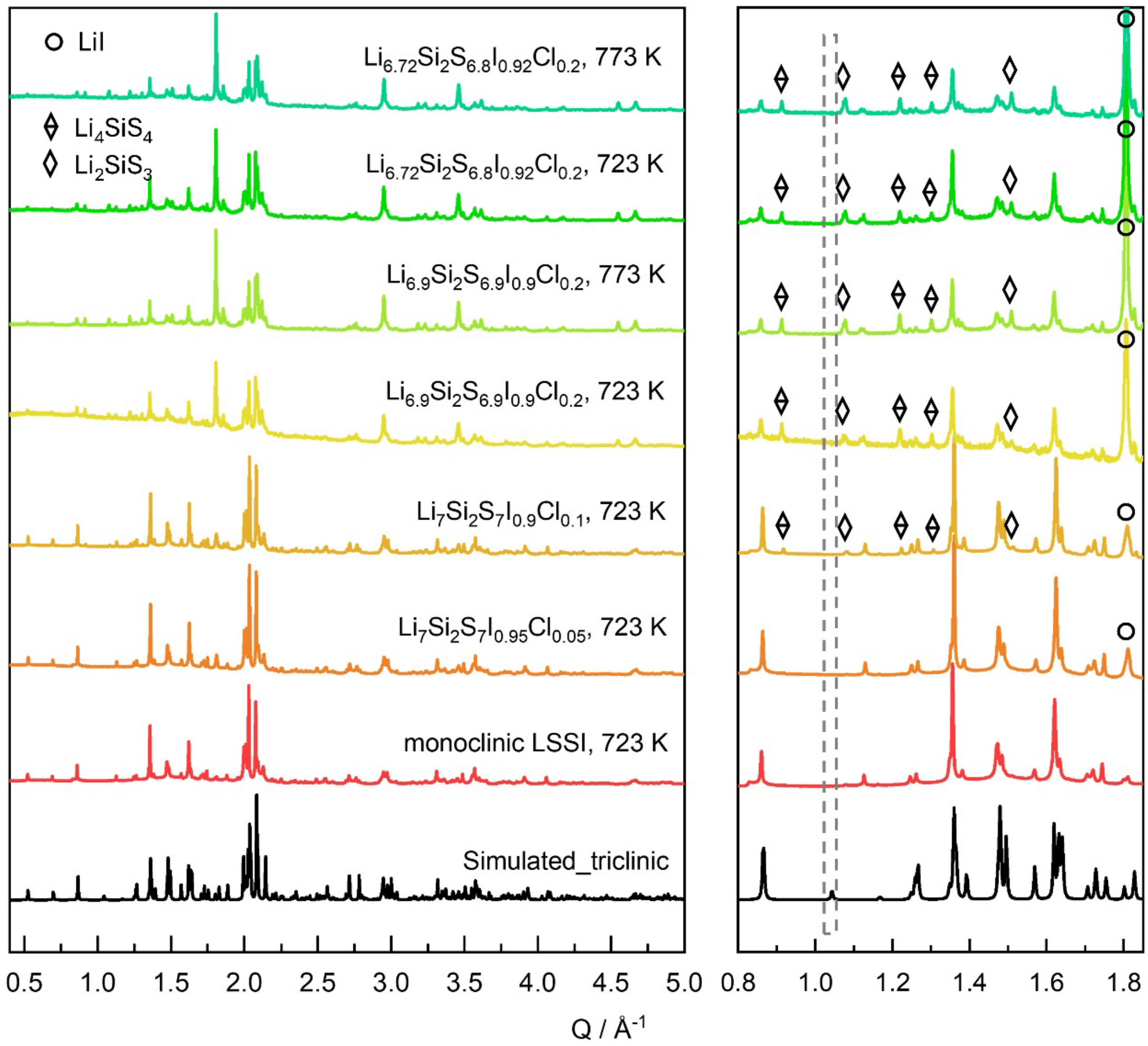

Figure S3. Synchrotron powder X-ray diffraction patterns of experimental Cl substitution samples compared to the simulated pattern of triclinic $Li_7Si_2S_7I_{0.89}Cl_{0.11}$. The different samples vary in composition and synthesis condition. The right panel displays the low angle PXRD patterns, with impurities Li_4SiS_4, Li_2SiS_3 and LiI marked. The dashed box highlights a unique peak of triclinic $Li_7Si_2S_7I_{0.89}Cl_{0.11}$ which is not present in the experimental patterns, indicating the absence of the triclinic phase in powder samples produced under the conditions tested.

Table S2. Crystal data, data collection and structure refinement parameters of triclinic $Li_7Si_2S_7I_{0.89}Cl_{0.11}$.

Radiation/Å	Mo Kα (λ = 0.71073)		
Crystal size/mm^3	0.05 × 0.06 × 0.07		
Crystal system, Space group	Triclinic, $P\bar{1}$		
Empirical formula	$Li_7Si_2S_7I_{0.89}Cl_{0.11}$		
Temperature/K	100.01(10)	241(3)	300.00(10)
a/Å	9.0345(3)	9.0486(5)	9.0428(6)
b/Å	6.0176(2)	6.0273(3)	6.0390(4)
c/Å	11.9234(4)	11.9663(6)	12.0347(6)
α/°	90.314(3)	90.135(4)	89.794(4)
β/°	91.048(3)	90.937(4)	90.905(5)
γ/°	90.677(3)	90.498(4)	90.480(5)
Volume/Å^3	648.06(4)	652.51(6)	657.10(7)
Z	2	2	2
ρ_{calc}/g cm^{-3}	2.286	2.265	2.252
μ/mm^{-1}	3.492	3.446	3.433
F(000)	420.0	419.0	420.0
2Θ range for data collection/°	3.416 to 60.628	3.404 to 61.098	3.384 to 60.37
Reflections collected/ Independent reflections	15615/3302 [R_{int} = 0.0407]	10174/3044 [R_{int}= 0.0318]	8973/3158 [R_{int}=0.0429]
Data/restraints/parameters	3302/28/211	3044/64/227	3158/72/248
Goodness-of-fit on F^2	1.053	1.043	1.038
Completeness (%)	100	98.0	98.5
Final *R* indexes [I≥2σ (I)]	R_1= 0.0209, wR_2= 0.0460	R_1= 0.0230, wR_2= 0.0391	R_1= 0.0275, wR_2= 0.0595
Final *R* indexes [all data]	R_1= 0.0256, wR_2= 0.0475	R_1= 0.0324, wR_2= 0.0410	R_1= 0.0355, wR_2= 0.0616
Largest diff. peak/hole / e Å^{-3}	0.66/-0.65	0.42/-0.42	0.83/-0.51
CSD Number	2429627	2429628	2429626

Table S3. Fractional atomic coordinates, site occupancy factors (*sof*) and equivalent isotropic displacement parameters U_{eq} ($Å^2$) of triclinic $Li_7Si_2S_7I_{0.89}Cl_{0.11}$ at 300 K. U_{eq} is defined as 1/3 of the trace of the orthogonalised U_{ij} tensor.

**Site**	**Wyck. Site**	***sof***	***x/a***	***y/b***	***z/c***	**U_{eq}**
I1|Cl1	2i	0.8873(17)|0.1127(17)	0.98983(2)	0.75142(3)	0.88611(2)	0.02632(8)
S1	2i	1	0.68019(8)	0.21170(12)	0.91514(6)	0.02387(17)
S2	2i	1	0.30494(8)	0.24469(12)	0.83563(6)	0.02432(17)
S3	2i	1	0.53688(8)	0.69941(11)	0.81215(6)	0.02462(18)
S4	2i	1	0.56009(8)	0.23262(11)	0.63680(5)	0.01909(16)
S5	2i	1	0.94371(8)	0.22371(11)	0.66759(5)	0.02117(16)
S6	2i	1	0.76527(8)	0.23619(12)	0.40833(6)	0.02386(17)
S7	2i	1	0.77024(8)	0.70581(11)	0.56869(6)	0.02293(17)
Si1	2i	1	0.52269(8)	0.35252(12)	0.80646(6)	0.01745(17)
Si2	2i	1	0.76995(8)	0.35904(12)	0.57160(6)	0.01671(17)
Li1	2i	0.920(19)	0.7594(7)	0.9311(10)	0.7499(5)	0.040(2)
Li2	2i	1	0.5061(6)	0.7728(11)	0.6129(5)	0.0484(17)
Li3	2i	0.943(19)	0.9417(9)	0.2898(13)	0.8710(5)	0.055(3)
Li4	2i	0.29(2)	0.415(3)	0.142(5)	1.0143(18)	0.037(4)
Li5	2i	0.29(2)	0.285(3)	0.827(4)	0.816(3)	0.049(6)
Li6	2i	0.311(19)	0.290(2)	0.644(4)	0.899(2)	0.042(5)
Li7	2i	0.415(16)	0.2932(17)	0.378(3)	1.0252(11)	0.041(4)
Li8	2i	0.38(2)	0.996(2)	-0.082(4)	0.5368(18)	0.045(5)
Li9	2i	0.54(2)	0.7997(17)	0.652(3)	0.3595(10)	0.051(5)
Li10	2i	0.587(18)	0.9904(13)	0.367(2)	0.3339(9)	0.053(4)
Li11	2i	0.46(2)	0.7577(17)	0.796(4)	0.3703(11)	0.045(5)
Li12	2i	0.19(2)	0.455(5)	0.076(8)	1.005(3)	0.050(7)
Li13	2i	0.24(2)	0.299(3)	0.832(6)	0.873(3)	0.042(6)
Li14	2i	0.148(14)	0.315(5)	0.218(8)	1.028(3)	0.041(6)
Li15	1b	0.26(4)	1	0	1/2	0.047(12)
Li16	2i	0.15(2)	0.287(4)	0.712(11)	0.854(5)	0.033(6)

Table S4. Anisotropic displacement parameters ($Å^2$) of triclinic $Li_7Si_2S_7I_{0.89}Cl_{0.11}$ at 300 K.

Site	U_{11}	U_{22}	U_{33}	U_{12}	U_{13}	U_{23}
I1|Cl1	0.02668(13)	0.02445(14)	0.02775(13)	0.00205(9)	-0.00273(9)	0.00148(8)
S1	0.0262(4)	0.0216(4)	0.0236(4)	0.0021(3)	-0.0031(3)	0.0030(3)
S2	0.0210(4)	0.0278(4)	0.0243(4)	-0.0027(3)	0.0027(3)	0.0002(3)
S3	0.0314(4)	0.0137(4)	0.0289(4)	0.0005(3)	0.0030(3)	-0.0008(3)
S4	0.0200(3)	0.0175(4)	0.0198(3)	-0.0028(3)	0.0021(3)	-0.0016(2)
S5	0.0214(4)	0.0190(4)	0.0231(4)	0.0028(3)	-0.0017(3)	0.0007(3)
S6	0.0257(4)	0.0268(4)	0.0191(4)	-0.0019(3)	0.0027(3)	-0.0037(3)
S7	0.0287(4)	0.0125(4)	0.0276(4)	0.0008(3)	0.0030(3)	0.0028(3)
Si1	0.0205(4)	0.0128(4)	0.0191(4)	0.0004(3)	0.0019(3)	0.0007(3)
Si2	0.0204(4)	0.0121(4)	0.0176(4)	-0.0001(3)	0.0013(3)	0.0013(3)
Li1	0.045(4)	0.031(4)	0.043(4)	-0.008(3)	0.003(3)	0.002(3)
Li2	0.023(3)	0.080(5)	0.041(4)	0.000(3)	-0.003(3)	0.009(3)
Li3	0.062(5)	0.065(5)	0.039(4)	-0.021(4)	0.003(3)	-0.010(3)
Li4	0.046(8)	0.041(7)	0.025(9)	0.000(5)	-0.003(5)	-0.001(5)
Li5	0.055(15)	0.042(6)	0.049(8)	-0.001(6)	0.001(7)	0.002(5)
Li6	0.040(11)	0.040(6)	0.046(8)	0.005(5)	0.010(6)	0.000(5)
Li7	0.049(8)	0.035(6)	0.040(7)	-0.004(5)	0.000(5)	-0.005(4)
Li8	0.050(12)	0.039(9)	0.045(8)	-0.006(8)	0.010(8)	-0.020(6)
Li9	0.052(8)	0.065(12)	0.037(7)	-0.018(8)	0.012(6)	-0.001(6)
Li10	0.049(7)	0.066(8)	0.045(7)	-0.003(6)	0.009(5)	-0.005(5)
Li11	0.045(9)	0.068(13)	0.022(7)	0.008(8)	-0.005(6)	-0.011(7)
Li12	0.054(10)	0.040(9)	0.056(15)	0.001(8)	0.016(9)	0.008(7)
Li13	0.036(14)	0.041(6)	0.048(8)	0.004(4)	0.004(7)	0.002(5)
Li14	0.044(9)	0.035(6)	0.044(13)	-0.005(5)	-0.001(6)	-0.005(5)
Li15	0.05(3)	0.041(11)	0.046(13)	-0.007(12)	0.009(12)	-0.018(10)
Li16	0.013(15)	0.041(6)	0.047(8)	0.005(4)	0.011(6)	0.001(5)

Table S5. Selected geometric Si^{4+} and Li^{+} coordination mode information for $Li_7Si_2S_7I_{0.89}Cl_{0.11}$ at 300 K.

Central atom	Length / Å		Central atom	Length / Å	
Si1	Si1—S1	2.1014(10)	Li8	Li8—S5	2.674(17)
	Si1—S2	2.1032(10)		Li8—S5	2.483(16)
	Si1—S3	2.099(1)		Li8—S6	2.44(2)
	Si1—S4	2.2009(10)		Li8—S7	2.44(2)
Si2	Si2—S4	2.1951(10)	Li9	Li9—S2	2.594(13)
	Si2—S5	2.1044(10)		Li9—S5	2.459(13)
	Si2—S6	2.1024(10)		Li9—S6	2.595(19)
	Si2—S7	2.0943(10)		Li9—S7	2.558(13)
Li1	Li1—I1|Cl1	2.851(6)	Li10	Li10—I1|Cl1	2.755(11)
	Li1—S1	2.726(6)		Li10—S5	2.535(12)
	Li1—S3	2.561(6)		Li10—S6	2.366(11)
	Li1—S4	2.894(6)		Li10—S7	2.487(12)
	Li1—S5	2.622(6)	Li11	Li11—S2	2.545(13)
	Li1—S7	2.578(6)		Li11—S4	2.877(15)
Li2	Li2—S3	2.449(5)		Li11—S5	2.749(16)
	Li2—S4	2.830(7)		Li11—S6	2.70(2)
	Li2—S6	2.464(5)		Li11—S7	2.448(13)
	Li2—S7	2.492(5)	Li12	Li12—S1	2.33(4)
Li3	Li3—I1|Cl1	2.824(7)		Li12—S1	2.46(4)
	Li3—I1|Cl1	3.288(8)		Li12—S2	2.64(4)
	Li3—I1|Cl1	2.989(7)		Li12—S3	2.59(5)
	Li3—S1	2.473(8)	Li13	Li13—I1|Cl1	2.84(3)
	Li3—S5	2.482(6)		Li13—S1	2.57(4)
Li4	Li4—S1	2.45(3)		Li13—S2	2.53(3)
	Li4—S1	2.73(3)		Li13—S3	2.43(3)
	Li4—S2	2.44(2)	Li14	Li14—I1|Cl1	2.96(5)
	Li4—S3	2.33(2)		Li14—S1	2.68(5)
Li5	Li5—I1|Cl1	2.84(3)		Li14—S2	2.32(4)
	Li5—S2	2.54(3)		Li14—S3	2.38(4)
	Li5—S3	2.42(3)	Li15	Li15—S5	2.4931(7)
	Li5—S6	2.76(3)		Li15—S5	2.4931(7)
Li6	Li6—I1|Cl1	2.79(2)		Li15—S6	2.7789(8)
	Li6—S1	2.41(2)		Li15—S6	2.7789(8)
	Li6—S2	2.54(2)		Li15—S7	2.8528(7)
	Li6—S3	2.50(2)		Li15—S7	2.8528(7)
Li7	Li7—I1|Cl1	2.889(15)	Li16	Li16—I1|Cl1	2.74(3)
	Li7—S1	2.592(15)		Li16—S1	2.82(6)
	Li7—S2	2.426(14)		Li16—S2	2.84(7)
	Li7—S3	2.513(14)		Li16—S3	2.32(4)

Table S6. Comparison of the occupied tetrahedral and octahedral environment types in the structures of LSSI, $Li_7Si_2S_7I_{0.89}Cl_{0.11}$ and $Li_7Si_{0.88}Ge_{1.12}I_7$ at 300 K.

Site type	$Li_7Si_2S_7I$ ($P2_1/n$)	$Li_7Si_2S_7I_{0.89}Cl_{0.11}$ ($P\bar{1}$)	$Li_7Si_{0.88}Ge_{1.12}S_7I$ ($P2_1/n$)
		hcp motif	
S_6	3× (Li4, Li12, Li8)	4× (Li4, Li8, Li12, Li15)	4× (Li4, Li8, Li12, Li16)
S_5I	1× (Li1)	1× (Li1)	1× (Li1)
S_3I	4× (Li7, Li10, Li14, Li15)	3× (Li7, Li14, Li10)	4× (Li7, Li10, Li14, Li15)
Total hcp	8	8	9
		sheared fcc-like motif	
S_6	3× (Li2, Li9, Li11)	3× (Li2, Li9, Li11)	3× (Li2, Li9, Li11)
S_5I	3× (Li5, Li6, Li13)	4× (Li5, Li6, Li13, Li16)	3× (Li5, Li6, Li13)
S_3I_3	1× (Li3)	1× (Li3)	1× (Li3)
Total sheared	7	8	7
Total Li^+ sites	15	16	16

Table S7. Comparison of the occupied tetrahedral and octahedral environment volumes in the structure of $Li_7Si_2S_7I_{0.89}Cl_{0.11}$ in the temperature range 100-300 K. Environments marked with an asterisk * are unoccupied but included for comparison purposes. Uncertainties on volumes were calculated using *Crystal Palace*.[13]

Interstitial site		Content	Anion set	Volume (Å^3)		
				**300 K**	**240 K**	**100 K**
		hcp motif				
$T1_{hcp}$	$T1a_{hcp}$-1	Si1	S_4	4.907(3)	4.882(2)	4.9119(18)
	$T1a_{hcp}$-2	Si2	S_4	4.890(3)	4.902(3)	4.8867(18)
$T2_{hcp}$	$T2a_{hcp}$-1	Li14	S_3I	7.879(4)	7.848(3)*	7.782(3)*
	$T2a_{hcp}$-2	Unoccupied	S_3I	7.655(3)*	7.569(3)*	7.484(3)*
	$T2b_{hcp}$-1	Li7	S_3I	8.312(4)	8.229(3)	8.130(3)
	$T2b_{hcp}$-2	Li10	S_3I	7.939(4)	7.866(3)	7.800(3)
$O1_{hcp}$	$O1a_{hcp}$-1	2 × Li4, 2 × Li12	S_6	26.665(13)	26.519(11)	26.366(10)
	$O1a_{hcp}$-2	2 × Li8, Li15	S_6	25.926(12)	25.761(11)	25.580(9)
$O2_{hcp}$	$O2a_{hcp}$	Li1	S_5I	25.641(9)	25.443(7)	25.261(6)
		Sheared fcc-like motif				
$O1_{fcc}$	$O1a_{fcc}$	Li9, Li11	S_6	27.547(9)	27.378(7)	27.200(6)
	$O1b_{fcc}$	Li2	S_6	26.574(8)	26.400(7)	26.249(6)
$O2_{fcc}$		Li5, Li6, Li13, Li16	S_5I	30.237(8)	29.982(7)	29.728(6)
$O3_{fcc}$		Li3	S_3I_3	31.115(8)	30.843(7)	30.566(6)

Table S8. Comparison of the occupied tetrahedral and octahedral environment volumes in the structure of $Li_7Si_2S_7I$ in the temperature range 100-300 K. Environments marked with an asterisk * are unoccupied but included for comparison purposes. Uncertainties on volumes were calculated using *Crystal Palace*.[13]

Interstitial site		Content	Anion set	Volume (Å^3)		
				**300 K**	**240 K**	**100 K**
		hcp motif				
$T1_{hcp}$	$T1a_{hcp}$-1	Si1	S_4	4.8784(14)	4.878(4)	4.903(4)
	$T1a_{hcp}$-2	Si2	S_4	4.8796(14)	4.875(4)	4.906(4)
$T2_{hcp}$	$T2a_{hcp}$-1	Li15	S_3I	7.9158(19)	7.868(5)*	7.827(4)*
	$T2a_{hcp}$-2	Li14	S_3I	7.7049(18)	7.662(4)*	7.613(4)*
	$T2b_{hcp}$-1	Li7	S_3I	8.364(2)	8.276(5)	8.206(5)
	$T2b_{hcp}$-2	Li10	S_3I	7.9822(19)	7.932(5)	7.895(4)
$O1_{hcp}$	$O1a_{hcp}$-1	2 × Li4, 2 × Li12	S_6	26.565(8)	26.485(18)	26.456(16)
	$O1a_{hcp}$-2	2 × Li8	S_6	26.180(8)	26.018(18)	25.982(17)
$O2_{hcp}$	$O2a_{hcp}$	Li1	S_5I	25.767(5)	25.588(12)	25.466(10)
		Sheared fcc-like motif				
$O1_{fcc}$	$O1a_{fcc}$	Li9, Li11	S_6	27.739(5)	27.584(12)	27.553(11)
	$O1b_{fcc}$	Li2	S_6	26.516(5)	26.386(11)	26.370(10)
$O2_{fcc}$		Li5, Li6, Li13	S_5I	30.184(5)	30.078(11)	30.002(10)
$O3_{fcc}$		Li3	S_3I_3	31.655(5)	31.309(10)	31.153(10)

Table S9. Comparison of anion-anion distances between I^-/Cl^- and adjacent anions in monoclinic LSSI and triclinic $Li_7Si_2S_7I_{0.89}Cl_{0.11}$ at 300 K.

Phase	Distance / Å		Phase	Distance / Å	
Monoclinic LSSI	I1—I1	4.1073(5)	Triclinic $Li_7Si_2S_7I_{0.89}Cl_{0.11}$	I1|Cl1—I1	4.0739(5)
	I1—S1	3.9956(5)		I1|Cl1—S1	3.9821(8)
	I1—S2	4.2800(5)		I1|Cl1—S2	4.1547(8)
	I1—S5	3.9266(6)		I1|Cl1—S5	3.8931(8)
	I1—S1	3.8159(6)		I1|Cl1—S1	3.8003(8)
	I1—S2	4.3562(6)		I1|Cl1—S2	4.3132(9)
	I1—S3	4.2115(5)		I1|Cl1—S3	4.1872(8)
	I1—S6	4.1742(6)		I1|Cl1—S6	4.2083(8)
	I1—S7	4.2724(6)		I1|Cl1—S7	4.2861(8)
	I1—I1	4.1073(5)		I1|Cl1—I1	4.0870(5)
	I1—S1	4.3073(5)		I1|Cl1—S1	4.2960(8)
	I1—S2	4.1680(5)		I1|Cl1—S2	4.2506(8)
	I1—S5	4.1388(5)		I1|Cl1—S5	4.1532(8)
Mean distance	4.143(156)		Mean distance	4.130(159)	

References

(1) Sharp, P. M.; Dyer, M. S.; Darling, G. R.; Claridge, J. B.; Rosseinsky, M. J. Chemically directed structure evolution for crystal structure prediction. *Phys. Chem. Chem. Phys.* **2020**, *22* (32), 18205-18218, 10.1039/D0CP02206C. DOI: 10.1039/D0CP02206C.
(2) Kresse, G.; Hafner, J. Ab initio molecular dynamics for liquid metals. *Phys. Rev. B* **1993**, *47* (1), 558-561. DOI: 10.1103/PhysRevB.47.558.
(3) Perdew, J. P.; Burke, K.; Ernzerhof, M. Generalized gradient approximation made simple. *Phys. Rev. Lett.* **1996**, *77* (18), 3865-3868. DOI: 10.1103/PhysRevLett.77.3865.
(4) Han, G.; Vasylenko, A.; Daniels, L. M.; Collins, C. M.; Corti, L.; Chen, R.; Niu, H.; Manning, T. D.; Antypov, D.; Dyer, M. S.; et al. Superionic lithium transport via multiple coordination environments defined by two-anion packing. *Science* **2024**, *383* (6684), 739-745. DOI: doi:10.1126/science.adh5115.
(5) Nosé, S. A unified formulation of the constant temperature molecular dynamics methods. *J. Chem. Phys.* **1984**, *81* (1), 511-519. DOI: 10.1063/1.447334.
(6) Waugh, J. S.; Fedin, E. I. Determination of hindered-rotation barriers in solids. *Sov. Phys. Solid State* **1963**, *4* (8), 1633-1636.
(7) Deng, Z.; Zhu, Z.; Chu, I.-H.; Ong, S. P. Data-Driven First-Principles Methods for the Study and Design of Alkali Superionic Conductors. *Chem. Mater.* **2017**, *29* (1), 281-288. DOI: 10.1021/acs.chemmater.6b02648.
(8) CrysAlis^{Pro} Oxford Diffraction/Agilent Technologies UK Ltd, Yarnton, England.
(9) Sheldrick, G. M. SHELXT - Integrated space-group and crystal-structure determination. *Acta Crystallogr. A* **2015**, *71*, 3-8, Article. DOI: 10.1107/s2053273314026370.
(10) Sheldrick, G. M. Crystal structure refinement with SHELXL. *Acta Crystallogr. C* **2015**, *71* (1), 3-8. DOI: doi:10.1107/S2053229614024218.
(11) Dolomanov, O. V.; Bourhis, L. J.; Gildea, R. J.; Howard, J. A. K.; Puschmann, H. OLEX2: a complete structure solution, refinement and analysis program. *J. Appl. Crystallogr.* **2009**, *42* (2), 339-341. DOI: doi:10.1107/S0021889808042726.
(12) Momma, K.; Izumi, F. VESTA 3 for three-dimensional visualization of crystal, volumetric and morphology data. *J. Appl. Crystallogr.* **2011**, *44* (6), 1272-1276. DOI: doi:10.1107/S0021889811038970.
(13) Angel, R. J.; Mazzucchelli, M. L.; Baratelli, L.; Schweinle, C. F.; Balić-Žunić, T.; Gonzalez-Platas, J.; Alvaro, M. Uncertainties of recalculated bond lengths, angles and polyhedral volumes as implemented in the Crystal Palace program for parametric crystal structure analysis. *Acta Crystallogr., Sect. A:Found. Adv.* **2025**, *81* (3), 202-210. DOI: doi:10.1107/S2053273325002682.
